# CIRBP promotes ferroptosis by interacting with ELAVL1 and activating ferritinophagy during renal ischaemia‐reperfusion injury

**DOI:** 10.1111/jcmm.16567

**Published:** 2021-06-10

**Authors:** Mingxing Sui, Da Xu, Wenyu Zhao, Hanlan Lu, Rui Chen, Yazhe Duan, Yanhua Li, Youhua Zhu, Lei Zhang, Li Zeng

**Affiliations:** ^1^ Department of Organ Transplantation Shanghai Changhai Hospital Shanghai China; ^2^ Department of Urology The Third Affiliated Hospital of Naval Medical University Shanghai China; ^3^ The Committee of Experts of China Organ Donation Beijing China

**Keywords:** acute kidney injury, CIRBP, ELAVL1, ferritinophagy, ferroptosis, ischaemia‐reperfusion

## Abstract

Renal ischaemia‐reperfusion (IR) is a major cause of acute kidney injury (AKI). Cold‐inducible RNA‐binding protein (CIRBP) may contribute to AKI because its deficiency protects against renal IR injury in a mechanism believed to involve ferroptosis. We aimed to investigate whether ferroptosis is associated with CIRBP‐mediated renal damage. The differential expression of CIRBP was examined in tubular epithelial (HK2) cells during hypoxia‐reoxygenation (HR) or in response to erastin, an inducer of ferroptosis. CIRBP expression was increased in response to HR or erastin in HK2 cells but the silencing of CIRBP inhibited HR and erastin‐induced ferroptosis together with ferritinophagy. We discovered an interaction between CIRBP and ELAVL1 using STRING software, which was verified through co‐immunoprecipitation and fluorescence colocalization assays. We found that ELAVL1 is a critical regulator in the activation of ferritinophagy and the promotion of ferroptosis. HR or erastin also induced the expression of ELAVL1. An autophagy inhibitor (hydroxychloroquine) or si‐ELAVL1 transfection reversed CIRBP‐enhanced ferritinophagy activation and ferroptosis in HK2 cells under HR. Injection of anti‐CIRBP antibody into a mouse model of IR inhibited ferroptosis and decreased renal IR injury in vivo. In summary, our results provide evidence that ferritinophagy‐mediated ferroptosis could be responsible for CIRBP‐enhanced renal IR injury.

## 1 INTRODUCTION

Renal ischaemia‐reperfusion (IR) injury caused by a disruption in the flow of blood is a major cause of morbidity and acute kidney injury (AKI).^1^, ^2^ The severity of AKI caused by IR is associated with the induction of inflammatory mediators but the exact mechanism is unclear.^3^, ^4^ Cold‐inducible RNA‐binding protein (CIRBP) is thought to be a modulator of inflammatory responses.^5^ Low levels of CIRBP are expressed constitutively but the expression is up‐regulated in response to certain conditions such as hypothermia, hypoxia, irradiation and haemorrhagic shock.^6^, ^7^, ^8^ Following IR, the levels of several proinflammatory factors such as interleukins are reduced in CIRBP‐deficient mice and the absence of CIRBP was found to attenuate the impact of IR.^9^, ^10^, ^11^

Ferroptosis is a caspase‐independent form of cell death that is associated with AKI and is known to contribute to renal IR injury.^12^, ^13^ Ferroptosis is induced by autophagy and is characterized by excessive levels of iron and lipid peroxidation.^14^, ^15^ Excessive lipid peroxidation is caused by the erastin‐induced depletion of the antioxidant glutathione (GSH).^16^ Erastin inhibits the production of GSH by binding to components in the cystine‐glutamate exchange system.^17^ Ferritinophagy occurs through the degradation of ferritin by the nuclear receptor coactivator 4 (NCOA4), an autophagy cargo receptor.^18^, ^19^ Ferroptosis is the predominant cause of cell death in oxidation damaged tissue but its exact role in IR injury is unclear.^20^

Recently, the RNA‐binding protein ELAVL1, also known as human antigen R, was found to be associated with ferroptosis in liver fibrosis.^21^ ELAVL1 regulates mRNAs containing AU‐rich elements (AREs) in their 3′‐untranslated regions.^22^ Increased ELAVL1 expression correlates with the activation of ferritinophagy, and autophagy is promoted by the interaction of ELAVL1 with the ARE of Beclin1 mRNA.^21^ Several mRNAs associated with IR injury and kidney disease contain AREs that are regulated by ELAVL1.^23^, ^24^ ELAVL1 has similar characteristics to CIRBP1 in response to stress. Both ELAVL1 and CIRBP1 are known to translocate from the nucleus to the cytoplasm during stress to regulate mRNA.^25^ Moreover, CIRBP1 is thought to enhance the expression of cycle E1 during cell proliferation by positively regulating ELAVL1 in breast cancer.^26^

In this study, we investigated whether ferroptosis is associated with CIRBP‐mediated renal damage, and the molecular mechanism involving CIRBP. The expression of CIRBP was examined in tubular epithelial cells (HK2 cells) under hypoxia‐reoxygenation (HR) or following treatment erastin, which induces ferroptosis inductor. In addition, ferroptosis and ferroptosis‐related events were detected in HK2 cells with CIRBP either silenced or overexpressed. The role of ELAVL1‐activated ferritinophagy in the effect of CIRBP was investigated through treatment with the autophagic inhibitor hydroxychloroquine (HCQ) and with ELAVL1 silenced in HK2 cells with CIRBP overexpressed. We determined whether ferritinophagy‐mediated ferroptosis was responsible for CIRBP‐enhanced renal IR injury. Our data may provide evidence for further clinical therapeutic studies in renal IR injury.

## 2 MATERIALS AND METHODS

### 2.1 Cell culture, transfection and HR treatment

Tubular epithelial HK2 cells were obtained from ATCC and cultured in Dulbecco’s modified Eagle medium with 10% foetal bovine serum and 1% penicillin‐streptomycin solution (HyClone) at 37°C and 5% CO_2_. When the cell density reached 80%, the cells were passaged. For the down‐regulation of CIRBP and ELAVL1, siRNA (si‐CIRBP, SC‐97329), ELAVL1 siRNA (sc‐35619, si‐ELAVL1) and control siRNA (si‐control) were obtained from Santa Cruz Biotechnology. The CIRBP overexpression vector pCDNA3.1‐CIRBP (NM_001300829.2) was constructed by Hanbio. Cells were transfected with Lipofectamine 3000 (Invitrogen) according to the manufacturer’s instructions. In HR experiments, HK2 cells were exposed to 6/12 hours of hypoxia (5% CO_2_, 1% O_2_ and 94% N_2_) followed by reoxygenation (5% CO_2_, 21% O_2_ and 74% N_2_). For erastin treatment, cells were treated with erastin (0, 2, 4, 6, 8, 10 and 15 μmol/L) for 24 hours.

### 2.2 Cell viability analysis

To assess viability, HK2 cells were seeded onto 96‐well plates (5 × 10^3^ cells/well). CCK8 reagents (10 μL; CCK8 kit, Dojindo) were added to each well and incubated at 37°C for 4 hours. Absorbance was read at 450 nm on a microplate reader (Bio‐Rad). Cell morphology was assessed using a phase‐contrast microscope (Olympus).

### 2.3 Quantitative reverse transcription‐PCR

Total RNA was extracted from homogenized HK2 cells and renal tissues using TRIzol reagent (Thermo Fisher Scientific) following the manufacturer’s instructions. Quantitative reverse transcription‐PCR (qRT‐PCR) was conducted using a cDNA reverse transcription system (Promega) and SYBR Green Master Mix (Life Technologies) using the following primers: β‐actin Forward 5′‐CACGATGGAGGGGCCGGACTCATC‐3′, Reverse 5′‐TAAAGACCTCTATGCCAACACAGT‐3′; Homo CIRBP Forward 5′‐TTTGGGTTTGTCACCTTTG‐3′, Reverse 5′‐CTGCCTGGTCTACTCGGAT‐3′; Homo ELAVL1 Forward 5′‐TAAGGTGTCGTATGCTCGC‐3′, Reverse 5′‐TTTTTGTTCTGGTTGGGGT‐3′. The relative quantification method $2^{‐\Delta \Delta C_{t}}$ was used to measure the level of gene expression.

### 2.4 Western blot analysis

Proteins were extracted from cells and tissues using RIPA lysis buffer containing phenyl methane sulphonyl fluoride (Sigma‐Aldrich). Protein content was measured using a BCA protein assay kit (Pierce), and 40 μg protein from each sample was separated on 12% SDS‐PAGE. The separated proteins were transferred to a polyvinylidene fluoride (PVDF) membrane (Millipore). PVDF membranes were blocked with 5% non‐fat milk in TBST buffer for 2 hours at room temperature. Blocked membranes were incubated with primary antibody (CIRBP, ELAVL1, ferritin heavy chain (FTH1), LC3, BECN1, and β‐actin, internal control) overnight at 4°C using the manufacturer’s (Abcam) recommended dilutions. NAOA4 antibody was from Santa Cruz Biotechnology. They were then incubated for 2 hours in HRP‐conjugated anti‐rabbit or anti‐mouse IgG at room temperature. Immunoreactive protein bands were detected with ECL substrate (Beyotime), and images were analysed using Image‐Pro Plus.

### 2.5 Biochemical assays

Lipid peroxidation levels in cell and tissue lysates were measured by determining MDA (malondialdehyde) levels with a Lipid Peroxidation (MDA) Assay Kit (Abcam) according to the manufacturer’s instructions. A Glutathione Assay Kit (Sigma‐Aldrich) was used to measure glutathione (GSH), and an Iron Assay Kit (Abcam) was used to measure iron levels in cell and tissue lysates according to manufacturer’s instructions. Intracellular levels of ROS (H_2_O_2_) were measured using the fluorescent dye DCFH‐DA (Sigma‐Aldrich) with excitation and emission at 485 and 535 nm, respectively.

### 2.6 Immunofluorescence

HK2 cells were fixed with 4% paraformaldehyde and blocked using goat serum for 30 min. They were incubated with primary antibody (BECN1, LC3B, NCOA4, ferritin and LAMP2) for 1 hour and then with fluorescent‐labelled secondary antibodies. Nuclei were stained with DAPI, and images were obtained with a fluorescent microscope (Olympus).

### 2.7 Co‐immunoprecipitation assay

For the Co‐immunoprecipitation (Co‐IP) assay, cells were lysed with RIPA buffer containing 10 mmol/L N‐ethylmaleimide and a mixture of mammalian protease inhibitors and phosphatase inhibitors (Sigma‐Aldrich). Then, the filters were incubated with either CIRBP or ELAVL1 antibody and protein A‐agar beads (Thermo Fisher Scientific) at 4°C for 1 hour to obtain an immunoprecipitated (IP) sample. The IP sample and 10 μg of the input proteins were analysed using Western blot and probed with either ELAVL1 or CIRBP antibody.

### 2.8 Transmission electron microscopy

HK2 cells and renal tissues were prepared for transmission electron microscopy (TEM) as described previously.^27^ Briefly, cells were fixed with 2.5% glutaraldehyde at room temperature for 1 hour and post‐fixed with 1% osmium tetroxide. They were dehydrated at room temperature in a graded series of ethanol and after resin penetration were embedded in epoxy resin and polymerized for 48 hours at 60°C. Embedded samples were cut into ultrathin sections and stained with uranyl acetate and lead citrate. Images were obtained on an HT7700 transmission electron microscope (Hitachi).

### 2.9 Animal model of IR

Male C57BL/6 mice weighing 20‐25 g were subjected to IR as described previously.^28^ Briefly, to induce ischaemia, renal pedicles were accessed through a midline abdominal incision and clamped for 30 min. Mice body temperature was maintained at 32°C throughout the procedure using a heat‐pad. Clamps were removed and the abdomen was closed after confirmation that blood flow had returned to the kidneys. Reperfusion was allowed to continue for 24 hours. IR mice were intravenously injected with anti‐IgG or anti‐CIRBP (10 mg/kg body weight). IR model mice were injected with anti‐IgG or anti‐CIRBP (10 mg/kg body weight), ferrostatin‐1 (Fer‐1, 5 mg/kg body weight; Santa Cruz), or vehicle after ischaemia (n = 5 for each group). Serum was collected for renal function (BUN and creatinine) detection, renal tissues were embedded in paraffin and 4 μm sections were used for haematoxylin‐eosin (HE), Periodic acid‐Schiff (PAS) staining, immunohistochemical staining (IHC) and TEM. Fresh tissues were used for western blot. All experiments involving animals were approved by the animal ethics committee of our institution and followed animal welfare guidelines.

### 2.10 HE and immunostaining

Haematoxylin‐eosin, IHC and PAS staining were conducted as described previously.^28^, ^29^ For HE staining, 4 μm sections were deparaffinized, hydrated and then stained HE and PAS stains. For IHC staining, sections were deparaffinized and blocked in goat serum. Sections were first incubated with primary antibody against CIRBP and ELAVL1 for 1 hour. They were then incubated with HRP‐conjugated secondary antibody (BD Biosciences) and visualized by diaminobenzidine. The relative level was semi‐quantified by Image Pro‐Plus (Bethesda).

### 2.11 Statistical analyses

All data are presented as the mean ± standard deviation (SD). Statistical analysis was conducted using SPSS version 13.0 software (SPSS). The Student’s *t* test was used for comparisons, and *P* <.05 was considered statistically significant.

## 3 RESULTS

### 3.1 CIRBP expression is increased under HR and ferroptosis in HK2 cells

**FIGURE 1:**
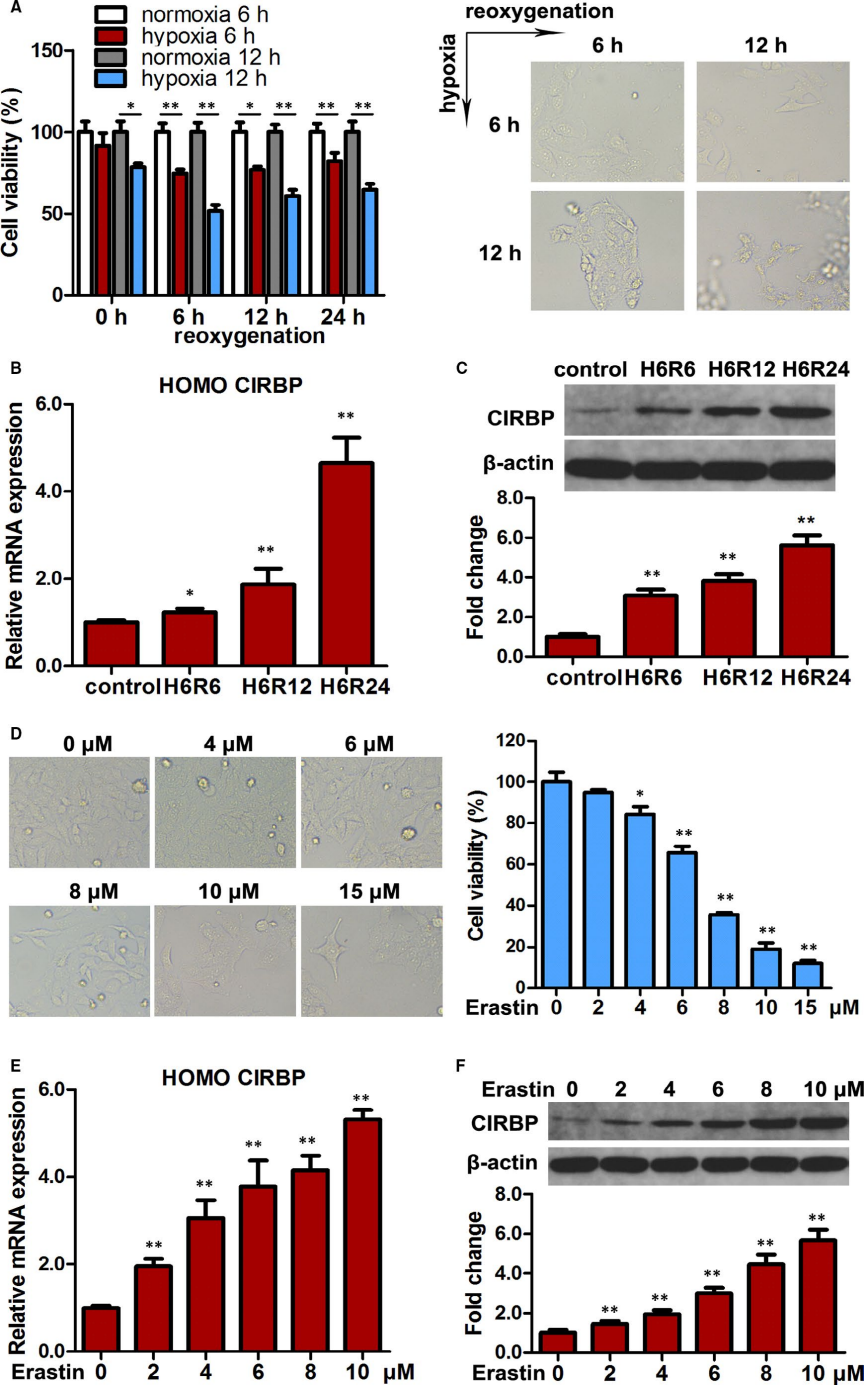
CIRBP expression is increased under hypoxia/reoxygenation (HR) and during ferroptosis in HK2 cells. A, HK2 cells subjected to normoxia and hypoxia for 6/12 h were then cultured for 24 h, and cell viability was measured. Representative images are shown (right panel). B, Relative mRNA expression of CIRBP detected by qRT‐PCR in HK2 cells cultured under HR. C, CIRBP protein levels in the indicated cells were assessed by western blotting. Representative blot (upper panel) and quantification (lower panel) are shown. β‐actin was used as loading control. D, Cell viability of HK2 cells treated with erastin (0‐15 μmol/L) for 24 h. Representative images are shown (left panel). E, Relative mRNA expression of CIRBP detected by qRT‐PCR in HK2 cells treated with erastin (0‐10 μmol/L) for 24 h. F, CIRBP protein levels in the indicated cells were assessed by western blotting, and representative blots (upper panel) and quantification (lower panel) are shown. Values are expressed as the mean ± SD. n = 3, ^*^ *P* < .05, ^**^ *P* < .01

To determine the characteristics of CIRBP expression under IR, we first examined its expression in HK2 cells subjected to HR and during ferroptosis. The cell viability of HK2 cells was reduced after 6 and 12 hours of hypoxia compared with cells grown under normal conditions (Figure 1A). The relative expression of CIRBP mRNA and protein levels increased after 6 hours of hypoxia and continued to increase during 24 hours reoxygenation (Figure 1B,C).

Erastin inhibits the cystine‐glutamate exchange system to trigger ferroptosis, a form of cell death that is dependent on intracellular iron levels.^30^ When HK2 cells were treated with erastin (0‐15 μmol/L) for 24 hours, cell viability decreased dose‐dependently (Figure 1D). Simultaneously, CIRBP mRNA expression and protein levels increased dose‐dependently (Figure 1E,F). These results indicate that the expression of CIRBP increases in human kidney epithelial cells that are affected adversely by HR and ferroptosis.

### 3.2 Suppression of CIRBP inhibits ferroptosis in HK2 cells

**FIGURE 2:**
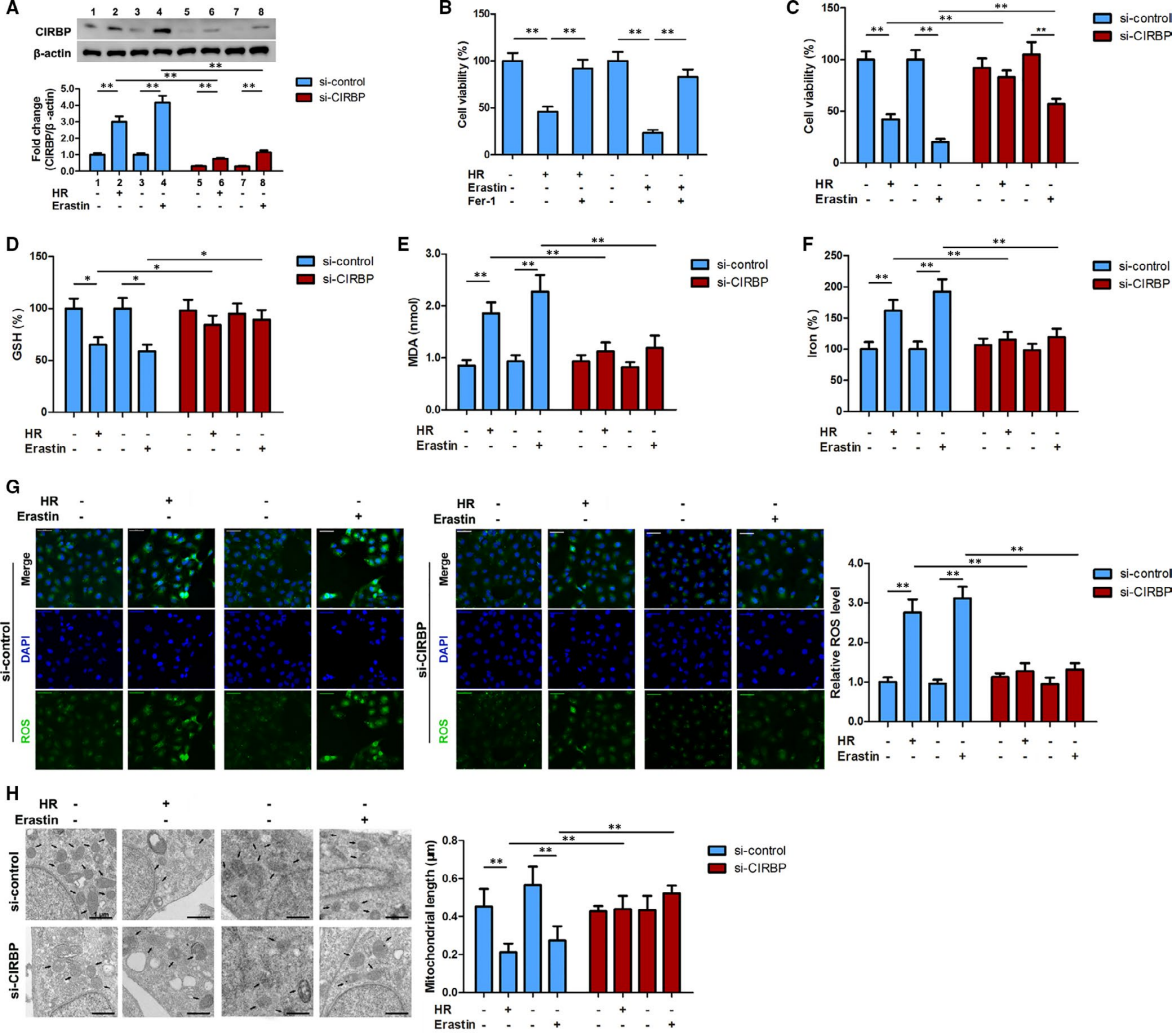
Silencing of CIRBP inhibits ferroptosis in HK2 cells. HK2 cells were transfected with CIRBP siRNA or control vector and then treated with hypoxia for 6 h and reoxygenation for 12 h (H6R12) or erastin (10 μmol/L, 24 h). A, The expression of CIRBP was detected by western blot in HK2 cells. B and C, Cell viability was measured by Cell counting kit‐8 (CCK‐8) assay. D‐G, Ferroptosis‐associated markers, GSH, MDA, iron, and ROS levels were assayed. H, Mitochondrial morphological changes were observed by transmission electron microscopy. Values are expressed as means ± SD. n = 3, ^*^ *P* < .05, ^**^ *P* < .01

To further investigate whether CIRBP may be involved in the ferroptosis of kidney cells, we silenced the expression of CIRBP in HK2 cells (A). The HK2 cells were then subjected to HR or treated with erastin (10 μmol/L, 24 hours) to induce ferroptosis. Levels of cell viability and the ferroptosis markers GSH (glutathione) depletion, MDA and iron accumulation were assessed (Figure 2B‐F). Cell viability was reduced significantly during HR and ferroptosis but reversed by ferroptosis inhibitors Fer‐1 treatment (8 μmol/L, 24 hours) (Figure 2B). The silencing of CIRBP appeared to improve viability, although it was still significantly lower than the control untreated HK2 cells during ferroptosis (Figure 2C). Levels of GSH depletion, MDA and iron were increased in HK2 cells subjected to HR. Similar results were found when HK2 cells were treated with erastin. However, when CIRBP was silenced in HK2 cells subjected to HR or treated with erastin, the levels of GSH, MDA and iron were not significantly different from the untreated controls (Figure 2D‐F). We also examined ROS levels in HK2 cells subjected to HR or treated with erastin with or without CIRBP expression (Figure 2G). ROS levels were significantly elevated in HK2 cells subjected to HR or treated with erastin compared with untreated control cells. However, when CIRBP is silenced, the ROS levels in cells subjected to HR or treated with erastin are not significantly different from the control cells. Mitochondrial morphological changes were observed under TEM (Figure 2H). The length of mitochondria was significantly reduced in cells subjected to HR or treated with erastin. However, when CIRBP is silenced there is no significant difference in the lengths of mitochondria between treated cells and the control. Overall, these results indicate that CIRBP is involved in the response to HR and ferroptosis in kidney epithelial cells and physiological changes in the cells are subdued by the suppression of CIRBP.

### 3.3 Activation of ferritinophagy is involved in CIRBP‐enhanced ferroptosis in HK2 cells

**FIGURE 3:**
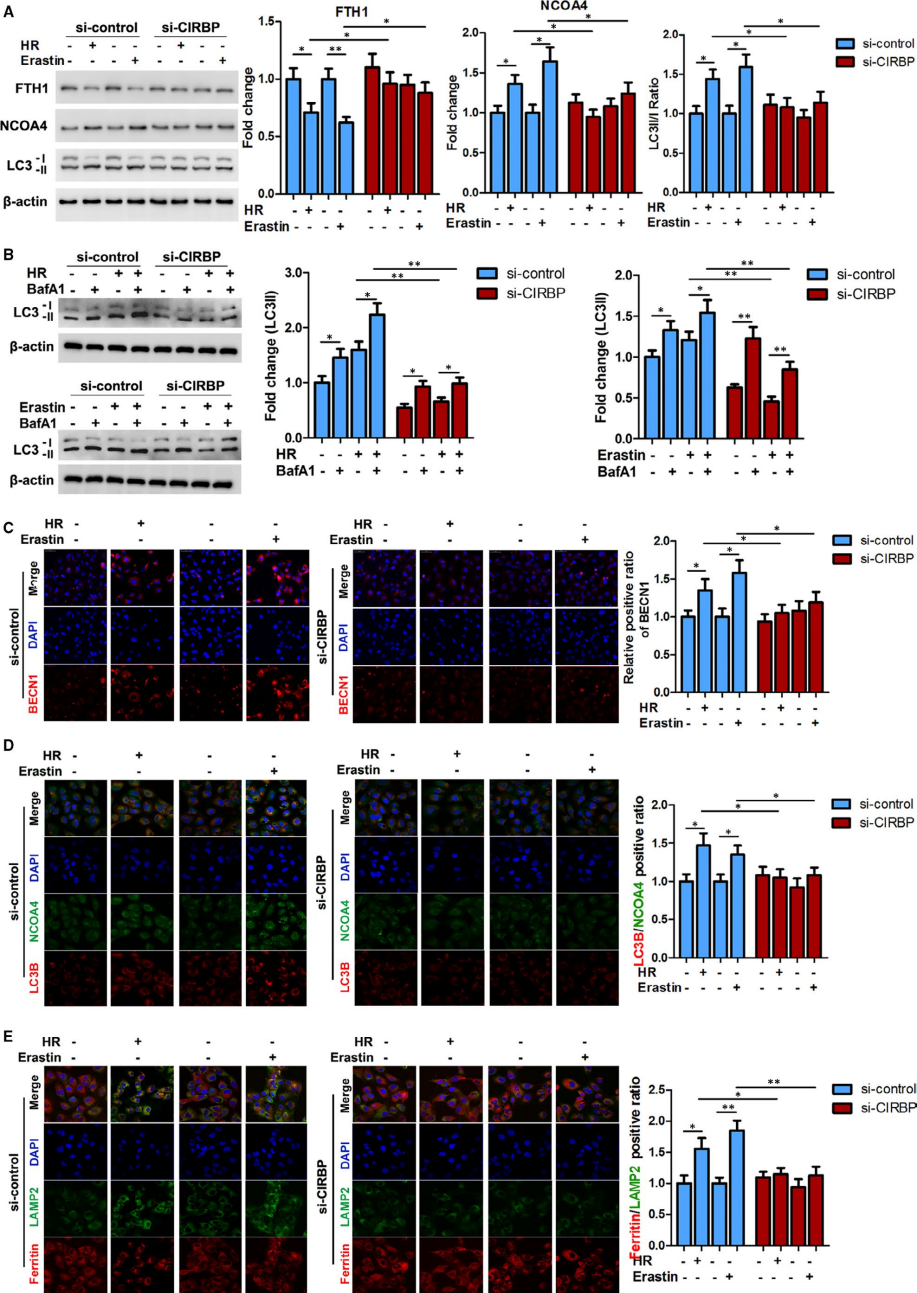
CIRBP‐enhanced ferroptosis is associated with ferritinophagy activation in HK2 cells. HK2 cells were transfected with CIRBP siRNA or control vector and then treated with hypoxia for 6 h and reoxygenation for 12 h (H6R12) or erastin (10 μmol/L, 24 h). A, Protein levels of FTH1, NCOA4, and LC3II/I were determined by western blotting; representative blots (left panel) and quantification (right panel) are shown. B, Representative immunoblotting and quantification data for LC3 II in the treated HK2 cells together with bafilomycin A1 (BafA1, 10 nmol/L) treatment. C, Representative immunofluorescence images of BECN1. Scale bar, 50 μm. D, Representative double‐immunofluorescence images of LC3B (red) and NCOA4 (green) by confocal microscopy. Magnification, 600×. Nuclei were stained with DAPI (blue). E, Representative double‐immunofluorescence images of ferritin (red) and LAMP2 (green) by confocal microscopy. Magnification, 600×. Nuclei were stained with DAPI (blue). Values are expressed as means ± SD. n = 3, *P < .05, **P < .01

To investigate the role of CIRBP in the response to HR and ferroptosis in greater detail, HK2 cells transfected with CIRBP siRNA or a control vector were subjected to HR or treated with erastin (10 μmol/L, 24 hours) and levels of ferritinophagy substrate‐FTH1, ferritinophagy receptor‐NCOA4, and the autophagy markers LC3II/I were determined by western blotting (Figure 3A). HR and erastin significantly lowered the levels of FTH1 in control cells but in cells with silenced CIRBP, FTH1 levels were not significantly different from the control. In contrast, levels of NCOA4 and LC3II/I were increased in HR and erastin treated HK2 cells but not when CIRBP is silenced. Enhanced LC3II accumulations were observed in each group together with lysosomal inhibitor bafilomycin A1 (BafA1, 20 nmol/L) treatment (Figure 3B), indicating a functionally intact flux of autophagy. These data suggest that ferritinophagy were increased in kidney cells undergoing HR and ferroptosis but the silencing of CIRBP prevented ferritinophagy. We also examined the expression of BECN1, LC3B/NCOA4 and ferritin/LAMP2 by immunofluorescence in HK2 cells with and without the expression of CIRBP (Figure 3C‐E). Levels of BECN1, an indicator of autophagy, were increased in response to HR and erastin but the silencing of CIRBP led to insignificant changes in the levels of BECN1 in response to HR and erastin. Similarly, ratios of LC3B/NCOA4 and ferritin/LAMP2 were elevated in response to HR and erastin when CIRBP was expressed but when the expression of CIRBP was silenced, the ratios were no different to control HK2 cells. These results demonstrate that CIRBP‐enhanced ferroptosis is associated with the activation of ferritinophagy in HK2 cells.

### 3.4 CIPRB interacts with ELAVL1

**FIGURE 4:**
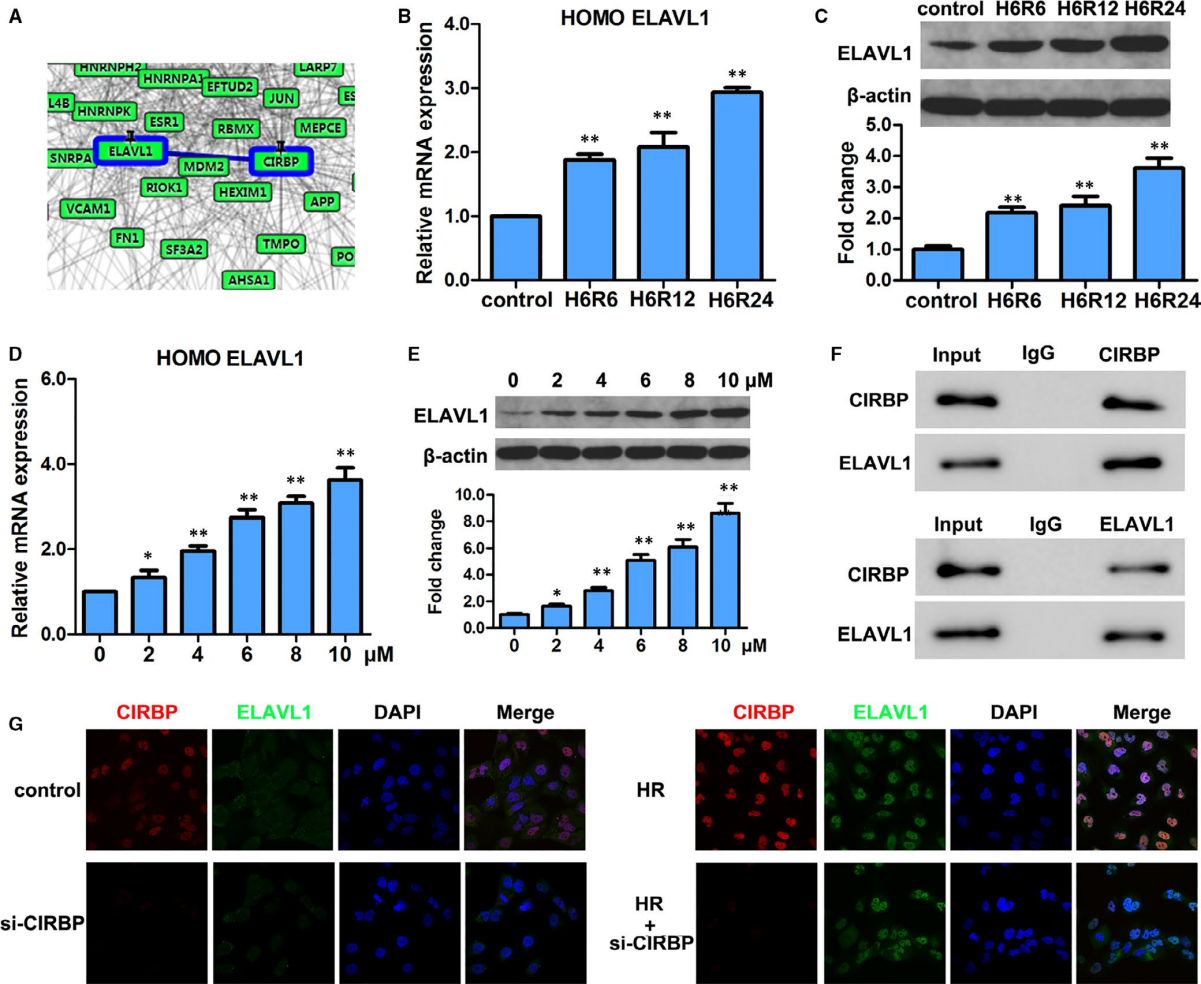
CIPRB interacts with ELAVL1. A, Interaction analysis of CIRBP and ELAVL1 by protein interaction software‐STRING. B, HK2 cells cultured under hypoxia/reoxygenation (HR) in Figure 1B were collected, and relative mRNA expressions of ELAVL1 were detected by qRT‐PCR. C, Western blot analyses of ELAVL1 in the indicated cells treated in Figure 1C, and the same gel blots of β‐actin were used as loading control. Representative blot (upper panel) and quantification (lower panel) are shown. D and E, mRNA and protein expression of ELAVL1 were detected by qRT‐PCR and western blotting in HK2 cells treated with erastin (0‐10 μmol/L) in Figure 1E and 1F, respectively. The data and gel blots of β‐actin in Figure 1E and 1F were also used as internal control here. F, Co‐IP assays of CIRBP and ELAVL1. G, Co‐localization of CIRBP and ELAVL1 analysed by fluorescence microscope. Magnification, 600×. Values are expressed as means ± SD. n = 3, *P < .05, **P < .01

Using STRING software, we discovered that CIPRB shared an interaction network with the stress‐related RNA‐binding protein ELAVL1 (Figure 4A). To determine whether ELAVL1 responded to HR, we measured its expression levels in HK2 cells subjected to HR and found a similar pattern to that of CIPRB. Following 6 hours of hypoxia, ELAVL1 mRNA expression and protein levels gradually increased as the length of reoxygenation increased (Figure 4B,C). As with CIPRB, the ELAVL1 mRNA expression and levels of protein increased with increasing concentration of erastin (Figure 4D,E). In addition, ELAVL1 was immunoprecipitated with CIPRB in a co‐IP assay (Figure 4F). Immunofluorescence analysis confirmed that they co‐localized in response to HR in HK2 cells (Figure 4G). Overall, these results suggest that ELAVL1 may regulate a response to HR and ferroptosis in an interaction with CIRBP.

### 3.5 ELAVL1 silencing reverses CIRBP‐mediated ferritinophagy activation

**FIGURE 5:**
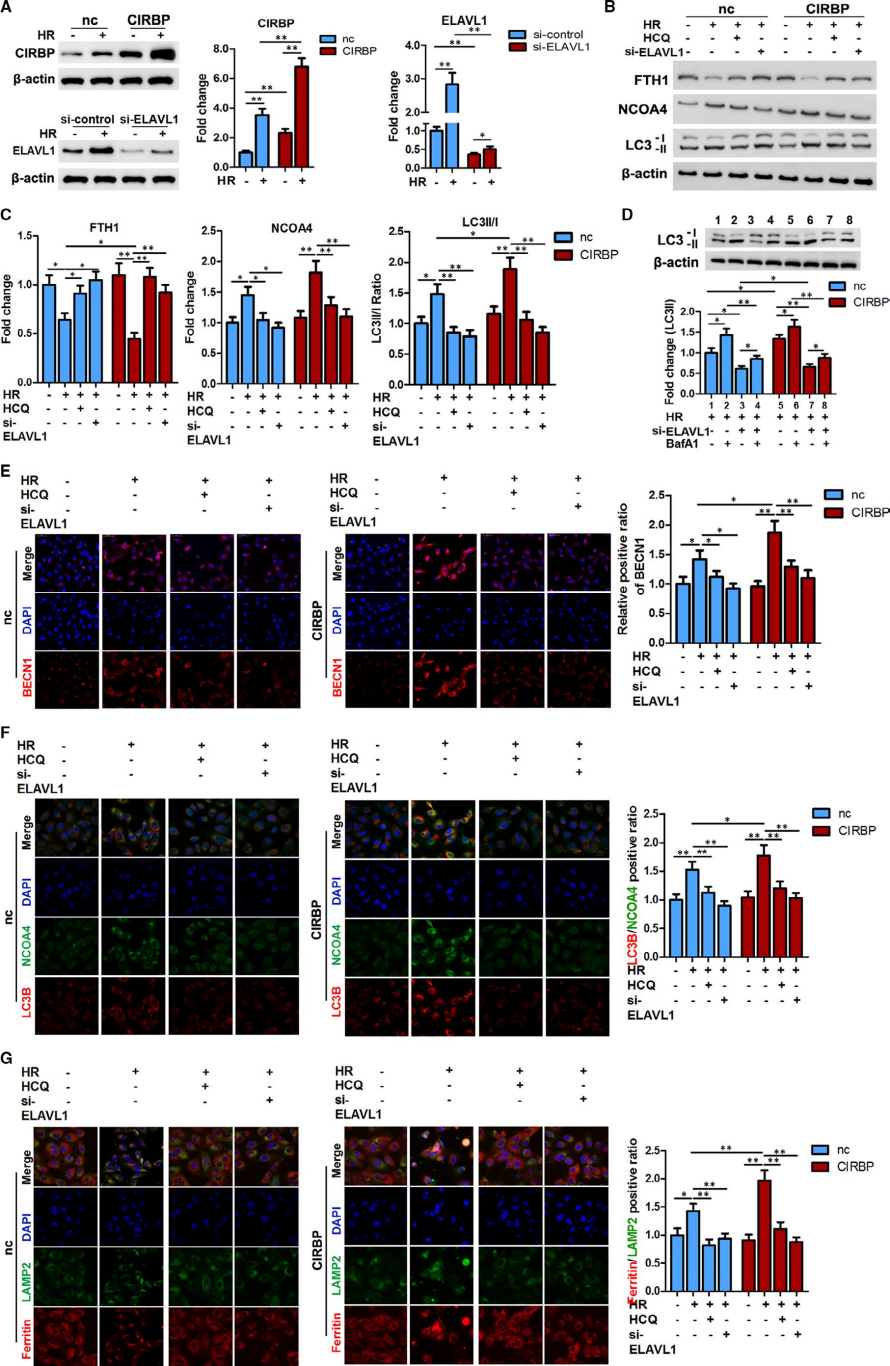
CIRBP induced ferritinophagy activation is inhibited by si‐ELAVL1. HK2 cells transfected with CIRBP or negative control (nc) vector were co‐transfected with si‐ELAVL1 or treated with hydroxychloroquine (HCQ, 50 μmol/L, 24 h), and then cultured under 6 h hypoxia and 12 h reoxygenation (H6R12). A, The expressions of CIRBP and ELAVL1 were confirmed after transfection. B, Representative blots of protein levels of FTH1, NCOA4, and LC3II/I. C, Quantification data of FTH1, NCOA4, and LC3II/I for each group. D, Representative immunoblotting and quantification data for LC3 II in the indicated cells together with BafA1 treatment. E, Representative immunofluorescence images of BECN1. Scale bar, 50 μm. F, Representative double‐immunofluorescence images of LC3B (red) and NCOA4 (green) by confocal microscopy. Magnification, 600×. Nuclei were stained with DAPI (blue). G, Representative double‐immunofluorescence images of ferritin (red) and LAMP2 (green) by confocal microscopy. Magnification, 600×. Nuclei were stained with DAPI (blue). The values are expressed as means ± SD. n = 3, *P < .05, **P < .01

To investigate the extent of ELAVL1 and CIRBP interaction in ferritinophagy, we assessed levels of relevant protein markers in HK2 cells subjected to 6 hours hypoxia and 12 hours reoxygenation with ELAVL1 silenced or treated with hydroxychloroquine (HCQ, 50 μmol/L, 24 hours), a lysosomal autophagic inhibitor that is associated with iron homeostasis.^31^ The level of FTH1 was reduced after HR especially when CIRBP was overexpressed (Figure 5A), whereas the levels of NCOA4 and the LC3II/I ratio were increased under the same conditions (Figure 5B,C), implying that HR increases the level of ferritinophagy with CIRBP contributing to increased levels. Meanwhile, BafA1 treatment further increased LC3II accumulation in the treated cells (Figure 5D). However, the use of HCQ or silencing the expression of ELAVL1 (Figure 5A) decreased the level of ferritinophagy after HR (Figure 5B,C). The decreased level of ferritinophagy was confirmed through immunofluorescence imaging using BECN1, LC3B/NCOA4, and ferritin/LAMP2 (Figure 5E‐G). These results demonstrate that the activation of CIRBP‐related ferritinophagy is inhibited by silencing the expression of ELAVL1.

### 3.6 Blocking ELAVL1‐activated ferritinophagy abolishes CIRBP‐enhanced ferroptosis

**FIGURE 6:**
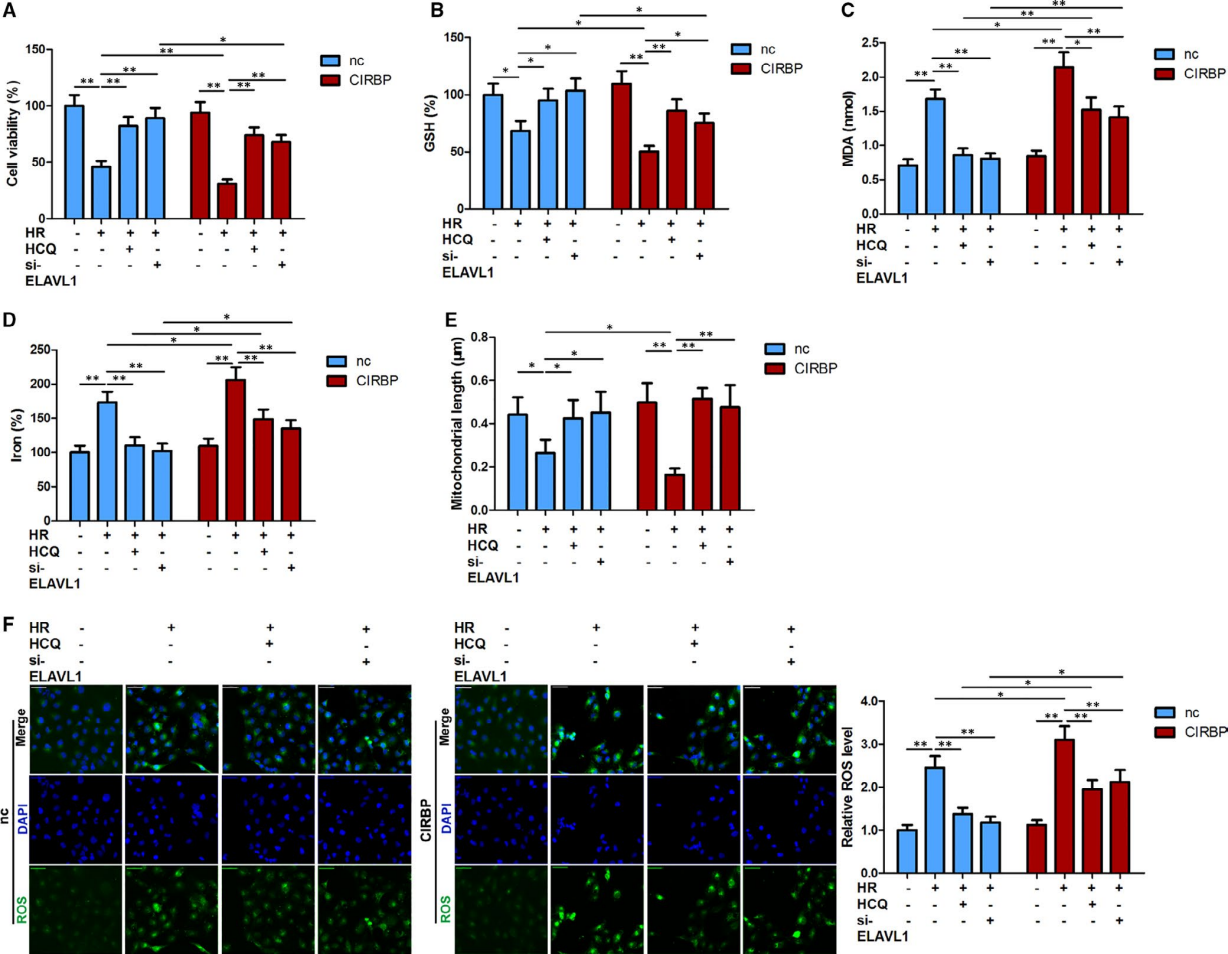
Ferritinophagy inhibition disrupted CIRBP‐enhanced ferroptosis. Cell viability (A), and ferroptosis associated markers, GSH, MDA content, iron, (B‐D) and ROS levels (F) were assayed in the indicated HK‐2 cells. E, Mitochondrial length was determined under transmission electron microscopy. Values are expressed as means ± SD. n = 3, *P < .05, **P < .01

In addition to the inhibition of ferritinophagy, blocking the expression of ELAVL1 led to the disruption of CIRBP‐enhanced ferroptosis. Cell viability was decreased and the levels of GSH were lower in HK2 cells after HR; however, when ELAVL1 is silenced or autophagy is inhibited by HCQ, cell viability and GSH level were increased (Figure 6A,B). In contrast, levels of MDA and iron were significantly higher after HR but were lowered when autophagy was inhibited by HCQ or the expression of ELAVL1 was blocked (Figure 6C,D). These results indicate that HR elevates ferroptosis but the inhibition of autophagy or silencing the expression of ELAVL1 reduces the level of ferroptosis. The overexpression of CIRBP resulted in higher levels of ferritinophagy following HR in HK2 cells but when levels of autophagy were inhibited or the expression of ELAVL1 was blocked the enhanced ferroptosis by CIRBP was abolished. Measurements of ROS levels and mitochondrial length substantiate the finding that blocking ELAVL1‐activated ferritinophagy lowers the level of CIRBP‐enhanced ferroptosis following HR in HK2 cells (Figure 6E,F).

### 3.7 CIRBP blockage inhibits ferroptosis and decreased renal IR injury *in* *vivo*

**FIGURE 7:**
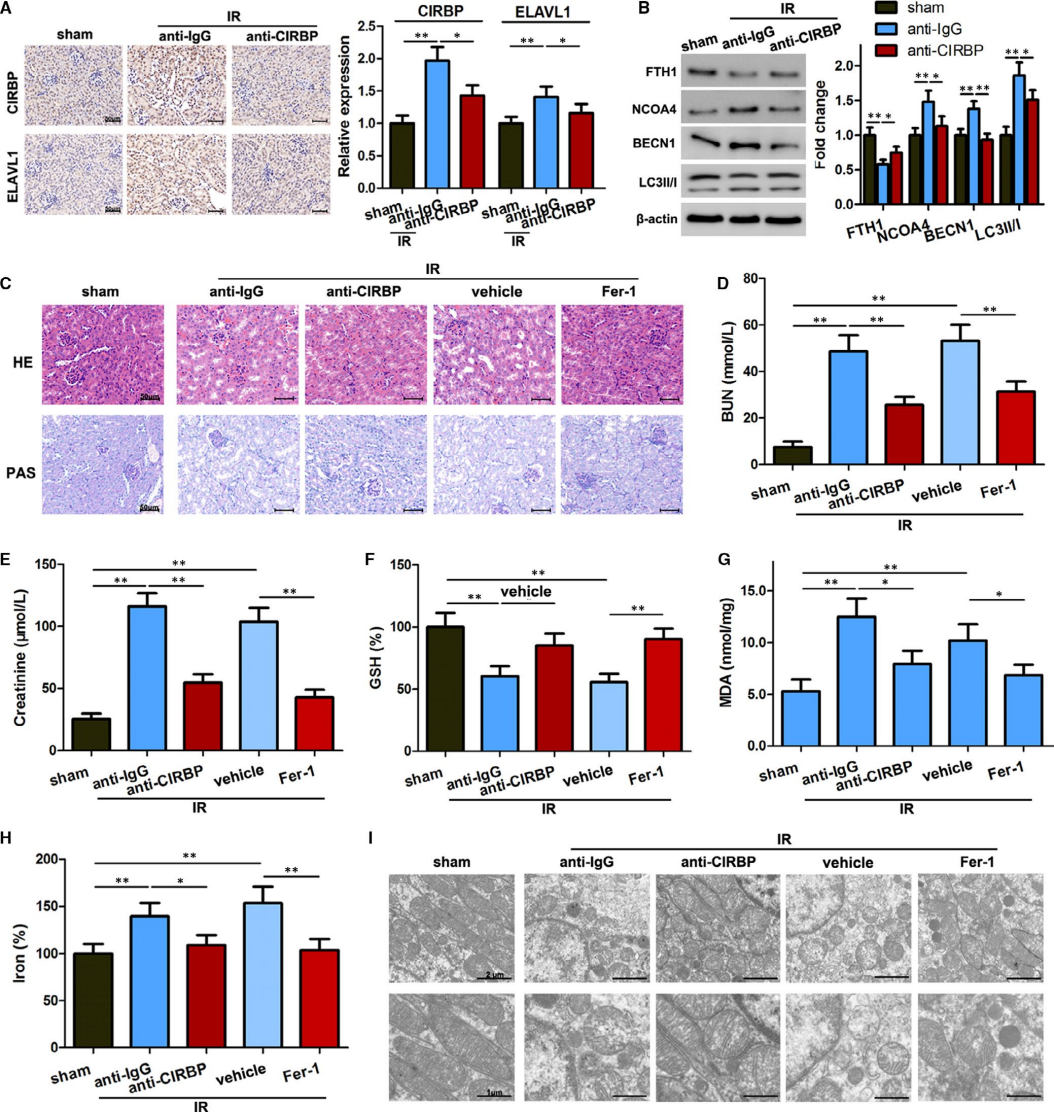
Anti‐CIRBP antibody inhibits ferroptosis and decreases renal ischaemia‐reperfusion (IR) injury in vivo. IR model mice were injected with anti‐IgG or anti‐CIRBP, ferrostatin‐1 (Fer‐1) or vehicle before reperfusion (n = 5 for each group). A, Protein levels of CIRBP and ELAVL1 were assayed using immunohistochemistry. Scale bar, 50 μm. B, Protein levels of FTH1, NCOA4, LC3II/I, and BECN1 in renal tissues. C, Representative images of HE and PAS staining. Scale bar, 50 μm. D and E, Serum BUN (D) and creatinine (E) levels after 24 h reperfusion. F‐H, GSH, MDA, and iron levels in renal tissues. I, Transmission electron microscopy images from each group of mice. Scale bars = 2 μm or 1 μm. Results are expressed as means ± SD. * *P* < .05, ** *P* < .01

Our results in vitro were validated in vivo through the use of a renal IR injury model in C57BL/6 mice. The mice were subjected to ischaemia for 30 minutes followed by reperfusion for 24 hours. Anti‐CIRBP or anti‐IgG was injected at reperfusion after ischaemia. Anti‐CIRBP significantly reduced the increased expression of CIRBP and ELVAL1 in IR model mice (Figure 7A) and seemed to attenuate the effects of IR on ferritinophagy as signified by levels of FTH1, NCOA4, LC3II/I and BECN1 in renal tissues (Figure 7B). Renal tissue samples confirmed that IR injury was alleviated when injected with anti‐CIRBP or Fer‐1. Levels of serum BUN and creatinine levels were found to be higher in IR model mice, which were reduced by an injection of anti‐CIRBP or Fer‐1 (Figure 7D,E). In addition, GSH levels were lower following IR but higher in mice injected with anti‐CIRBP, whereas the opposite occurred with levels of MDA and iron (Figure 7F‐H). There are fewer mitochondria in the cells of untreated IR model mice, which are reduced in size with less prominent mitochondrial cristae (Figure 7I). Overall, our results indicate that anti‐CIRBP antibody inhibits ferroptosis and decreases renal IR injury in vivo.

## 4 DISCUSSION

Cold‐inducible RNA‐binding protein is known to be up‐regulated in cells under hypoxia and in animal models following injuries relating to ischaemia.^32^, ^33^ The deficiency or blockade of CIRBP is reported to lessen the impact of IR injury with mechanisms that are proposed to involve the suppression of oxidative stress and proinflammatory responses.^7^, ^10^, ^11^ For instance, a polypeptide derived from CIRBP was found to bind to a TLR4 coreceptor to block the function of CIRBP and attenuate renal IR injury in a murine model.^34^ In addition, Godwin et al^11^ proposed that CRIBP mediated the inflammatory response in hepatic IR. They found that blocking the expression of CRIBP with anti‐CIRP antibody in a mouse model of IR reduced inflammation and cell apoptosis and improved the survival rate considerably. Ferroptosis has also been implicated in the pathogenesis of renal I/R injury.^4^, ^35^ Linkerman et al^35^ demonstrated that iron‐dependent ferroptosis was responsible for the necrosis of renal tubules in models of IRI and oxalate crystal‐induced AKI. In this study, we investigated whether ferroptosis is associated with CIRBP‐mediated renal damage and the molecular mechanisms involved.

In agreement with previous research,^36^ we found that CRIBP was up‐regulated in kidney tubular epithelial cells following HR. We also found that CRIBP was up‐regulated after the addition of erastin, a protein that triggers ferroptosis. When CRIBP is up‐regulated, the viability of cells is reduced implying that CRIBP could influence cell death. Therefore, we silenced the expression of CRIBP and measured parameters associated with ferroptosis in kidney tubular epithelial cells following HR. The accumulation of iron‐dependent ROS and a reduction in the size of mitochondria are characteristics of ferroptosis.^37^, ^38^ Increased ROS levels and a reduction in the number and size of mitochondria accompanied up‐regulated levels of CRIBP. However, when CRIBP was silenced, ROS levels and mitochondria were similar to those in untreated control cells. Other ferroptosis characteristics, such as GSH depletion, MDA and iron accumulation, also indicated that the down‐regulation of CRIBP was associated with reduced levels of ferroptosis.

After further investigation, we established that the induction of ferroptosis was associated with the activation of ferritinophagy. ELAVL1 is believed to regulate ferroptosis through the activation of ferritinophagy.^21^ Zhang et al^21^ found that the up‐regulation of ELAVL1 was accompanied by increased levels of ferritinophagy and ferroptosis in human hepatic stellate cells and they propose that ELAVL1‐dependent ferroptosis may be a potential target in the treatment of liver fibrosis. Similarly, high levels of CRIBP expression are believed to contribute to IR‐induced liver injury.^11^ Godwin et al^11^ were able to reduce IR injury in a mouse model of hepatic ischaemia using anti‐CIRBP antibody and observed a dramatic decrease in the levels of apoptosis and nitrosative stress. There are similarities between the behaviour of CRIBP and ELVAL1 in response to stress, suggesting that they may interact in response to hypoxia. We found that CRIBP and ELVAL1 interacted in response to HR and erastin. CIRBP‐related ferritinophagy was inhibited by silencing ELVAL1. Furthermore, we adopted a similar approach to Godwin et al^11^ and assessed whether anti‐CIRBP antibody would impact the level of renal injury in a mouse model of IR. We found that mice injected with anti‐CIRBP had lower levels of CIRBP and ELAVL1 and that ferritinophagy and ferroptosis were reduced in renal tissue of mice injected with anti‐CIRBP.

Our results add to the growing evidence that implicates ferritinophagy and ferroptosis in kidney injury following IR.^15^, ^35^, ^39^ Linkermann et al^35^ found that ferroptosis mediates post‐ischaemic renal necrosis in murine models of IR and proposed that ferroptosis could be therapeutically targeted by ferrostatins. Friedmann Angeli et al^39^ discovered that the suppression of ferroptosis led to higher levels of GSH and prevented lipid‐oxidation‐induced acute renal failure and cell death. Similarly, we found that levels of GSH were higher when either CIRBP or ELAVL1 were suppressed and lower when ferroptosis was increased. Recently, Jiang et al^40^ found that pachymic acid increased levels of GSH expression and lowered ferroptosis in a murine model of IR. They proposed that a possible mechanism could be through the activation of NRF2.

To conclude, CIRBP promotes ferroptosis by interacting with ELAVL1 and activating ferritinophagy during renal IR injury. Our results provide experimental evidence that ferritinophagy‐mediated ferroptosis is responsible for CIRBP‐enhanced renal IR injury. Our research provides the basis for further clinical therapeutic studies of renal IR injury.

## CONFLICT OF INTEREST

All authors declare that there are no conflicts of interest.

## AUTHOR CONTRIBUTIONS

**Mingxing Sui:** Data curation (equal); Formal analysis (equal); Funding acquisition (equal); Investigation (equal); Methodology (equal); Resources (equal); Writing‐original draft (equal); Writing‐review & editing (equal). **Da Xu:** Data curation (equal); Investigation (equal); Methodology (equal); Resources (supporting); Software (supporting); Writing‐original draft (equal); Writing‐review & editing (equal). **Wenyu Zhao:** Investigation (equal); Methodology (equal); Project administration (supporting); Resources (supporting); Software (equal); Writing‐review & editing (supporting). **Hanlan Lu:** Methodology (supporting); Project administration (supporting); Resources (equal); Software (supporting). **Rui Chen:** Project administration (supporting); Resources (equal); Software (supporting). **Yazhe Duan:** Methodology (supporting); Project administration (supporting); Resources (supporting); Software (supporting). **Yanhua Li:** Investigation (supporting); Methodology (supporting); Project administration (supporting); Software (supporting); Validation (supporting). **Youhua Zhu:** Formal analysis (supporting); Investigation (supporting); Methodology (supporting); Resources (supporting). **Lei Zhang:** Conceptualization (equal); Resources (equal); Supervision (equal); Writing‐original draft (supporting); Writing‐review & editing (supporting). **Li Zeng:** Conceptualization (lead); Funding acquisition (lead); Methodology (supporting); Supervision (lead); Writing‐original draft (supporting); Writing‐review & editing (supporting).

## Data Availability

The data that support the findings of this study are available from the corresponding author upon reasonable request.
